# Comparing access to orphan medicinal products in Europe

**DOI:** 10.1186/s13023-019-1078-5

**Published:** 2019-05-03

**Authors:** Bernarda Zamora, Francois Maignen, Phill O’Neill, Jorge Mestre-Ferrandiz, Martina Garau

**Affiliations:** Office of Health Economics, Southside, 7th Floor, 105 Victoria Street, London, SW1E 6QT UK

**Keywords:** Orphan drugs, Access, HTA, Reimbursement

## Abstract

**Objectives:**

The primary objective of this study was to compare the availability and access of orphan medicinal products (OMPs) in the devolved nations in the United Kingdom (UK), France, Germany, Italy and Spain. Availability is defined as the possibility to prescribe OMPs. Access refers to their full or partial reimbursement by the public health service.

**Methods:**

Data were collated on: marketing authorisations, Health Technology Assessment (HTA) decisions, commissioning, and reimbursement decisions, and respective dates of these events for all the OMPs centrally authorised. Indicators of availability of and access to OMPs were calculated in each country and compared.

**Results:**

We found that since the implementation of the OMPs Regulation in 2000 to end of May 2016, 143 OMPs obtained a marketing authorisation in the European Union. These OMPs are most widely accessible in Germany and France. In the other countries between 30 and 60% of OMPs are reimbursed. In particular in England, less than 50% of centrally authorised OMPs are routinely funded by the NHS, with one-third of these recommended by NICE. In Germany reimbursement is automatically granted to all medicines which receive a marketing authorisation, immediately after authorisation – but since 2011, there is an evaluation and potentially a pricing negotiation between companies and sickness funds (third party payers). In the other countries, the shortest time from authorisation to a reimbursement decision is observed in Italy and France where it takes 18.6 and 19.5 months respectively on average.

**Conclusions:**

Marketing authorisation granted to OMPs is only the first step, as medicines reach patients when reimbursement decisions are implemented by national health systems (this applies to non-OMPs too). We found that more than a half of centrally authorised OMPs were available in the five selected countries, but that access to patients was further restricted by different national reimbursement policies, especially in the UK, Italy and Spain.

**Electronic supplementary material:**

The online version of this article (10.1186/s13023-019-1078-5) contains supplementary material, which is available to authorized users.

## Introduction

The European Union (EU) Regulation (EC) No 141/2000 on orphan medicinal products (OMPs) stated that “patients suffering from rare conditions should be entitled to the same quality of treatment as other patients”. This principle was reinforced in the June 2009 Council Recommendation on action in the field of rare diseases. The additional incentives for companies to develop OMPs, introduced by European regulation, is only the first step, given patient access is determined by the national healthcare systems through their processes of health technology assessment (HTA), pricing, financing, and clinical practice. The aim of this paper is to compare access to OMPs across the five major European markets: Germany, the UK, France, Italy, and Spain, including a comparison of England, Scotland and Wales within the UK.

To assess differences across countries on access to OMPs we defined first a benchmark of potentially available OMPs: these consist of those centrally authorised by the European Commission (EC) after receiving a positive opinion from the European Medicines Agency (EMA). Secondly, some indicators are defined to inform on access as a proxy of the percentage of patients who are effectively treated with that drug within the public health service. We present two indicators separately for “availability” and “access”. The indicator of “availability” is intended to measure the presence of the OMPs in a particular country so that it can be prescribed and dispensed in hospitals and pharmacies, independently of the financing source. The indicator of “access” takes into account affordability for the patient, by considering OMPs which are reimbursed by the public health system. The time elapsed between central authorisation (by EMA) and reimbursement is presented as an additional indicator of access.

Analyses on access to OMPs in Europe have been reported in recent literature covering different set of countries and of OMPs yet coinciding in the main message of strong variation in access to OMPs between European countries. However, this variability in access among countries is comparable to non-orphan drugs, although mean access, as measured by the average number of medicines with positive utilisation, is larger for non-orphans than for orphan drugs [13].

Annemans et al. [2] highlight pricing and reimbursement (P&R) decisions, which are made at country level, as the main hurdle for OMP uptake and document the consequent variation in the reimbursement of OMPs across European countries. The European Organisation for Rare Diseases (EURORDIS) presented results on access to centrally authorised OMPs in a large number of European countries. The 2006 survey considered 22 OMPs in 28 countries [3] including indicators of number of reimbursed OMPs and time from EC authorisation to reimbursement. A more recent analysis also from EURORDIS Le Cam [4] compared access to 60 OMPs across 10 countries showing that the percentage of available OMPs in a country, is a close indicator of the percentage of patients treated according to prevalence of the indicated rare diseases. According to Le Cam [4] average access to OMPs for the major European countries was estimated in 2010 at 70% of patients or two thirds of centrally authorised OMPs. Other studies comparing access across EU countries as the number of reimbursed OMPs out of a set of centrally authorised OMPs include Garau and Mestre-Ferrandiz [9], Trama et al. [16], Orofino et al. [11], de Varax et al. [5] which also presents data on time elapsed between central authorisation and reimbursement, and [10] which compares HTA decisions and reimbursement for 101 centrally authorised OMP in 8 European countries. A different indicator of access based on utilisation (volume sales per 100,000 habitants) is used in [12] to compare access to 17 centrally OMPs across 23 European countries. Similarly, Detiček et al. [6] use sales data within a 1-year period as indicator to compare access of 112 OMPs in 22 European countries.

This brief summary of the literature suggests that (1) there is limited evidence about availability and access to OMPs in Europe; and (2) the evidence actually suggests different results, depending on the study, and specially depending on the choice of indicators defined either from HTA and P&R decisions or from observed sales. The purpose of this study aligns with a definition of access defined from the perspective of P&R decisions which give equal access to all patients specified in the marketing authorisation of OMPs. In other words, we aim at offering a comprehensive look at this issue (both in terms of number of OMPs and delays), for a full list of OMPs, for the EU5 countries – bearing in mid the data limitations outlined at the end of the paper, and contradictions with studies based on sales data.

## Pricing and reimbursement policies

The interpretation of results must account for differences in P&R polices in the selected countries. UK, Italy and Spain have tax-based healthcare systems with budget allocations and in some cases HTA delegated to regions. France and Germany have social insurance-based healthcare with HTA and P&R centralised at national level. Specific considerations for orphan drugs are included in HTA evaluations and consequent coverage decisions in France, Germany, England, and Scotland [14].

In France, once an HTA recommendation is issued by the Haute Autorité de la Santé (HAS), the price of the medicine is subsequently subject to negotiations between the pharmaceutical company and the Ministry of Health. HAS issues two different ratings, one on the Service Médical Rendu (SMR - absolute therapeutic value) and one on the Amélioration du Service Médical Rendu (ASMR or added therapeutic value compared to the standard of care provided by the French healthcare system at the time of the appraisal). The SMR rating defines the reimbursement rate: 0, 15, 35, 65% or 100%. For OMPs with budget impact lower than €30 m, ASMR is considered proven, granting full reimbursement. OMPs can be fully reimbursed before central authorisation via the Temporary Authorisation of Use on a named patient basis (nominal ATU) or for all patients for a given indication (cohort ATU). The same can apply to OMPs to treat chronic disabling diseases (Affections de Longue Durée or ALD).

HTA process was introduced in Germany with the Act on the Reform of the Market for Medical Products (AMNOG) of 22 December 2010 affected price negotiations of OMPs. Before the inception of the AMNOG in 2011, all OMPs were automatically reimbursed in Germany without requiring evidence of therapeutic value (additional benefit). The AMNOG introduced HTA and more evidentiary requirements for OMPs to prove additional benefit, based on a ranking system. There are some exemption rules which grant automatic full reimbursement at the ex-factory price set by the manufacturer for many OMPs without requiring HTA evaluation. These exemption rules include a ceiling on annual budget impact of €50 m, and apply to active ingredients available before the AMNOG. Those OMPs with a budget impact above €50 m are subject to cost-benefit analysis by the independent Institute for Quality and Efficiency in Health Care (IQWiG), followed by a decision on “additional benefit” by the Federal Joint Committee (G-BA). During the G-BA benefit assessment process, these OMPs are fully reimbursed at the price set by the manufacturer; once published, the G-BA decision on additional benefit affects price negotiations between the manufacturer and the Statutory Health Insurance Funds. In this sense, reimbursement is considered automatic since HTA is not a pre-requisite for reimbursement in Germany [1].

In the UK, the three HTA agencies in the UK adopted special assessment criteria for OMPs. The National Institute for Health and Care Excellence (NICE) set up a specific programme for ultra-orphan drugs (Highly Specialised Technologies (HST)), although to date the programme has only appraised a limited number of interventions. The rest of OMPs (non ultra-orphan) are reviewed via the standard Technology Appraisal (TA) programme with similar methods applied to interventions for more common diseases. The Scottish Medicine Consortium (SMC) evaluations include orphan and ultra-orphan modifier criteria, and the Welsh agency All Wales Medicines Strategy Group (AWMSG) also includes some additional criteria to consider severity and unmet need. We note that nominative prescription of any available OMP from an NHS doctor provides automatic right to reimbursement to the patient, but we focused on routine funding decisions to measure access.

NICE makes recommendations on the appropriate use of medicines in the NHS which, following a positive recommendation, lead to mandatory funding at the local level. This funding should be made available within three months from the date that the Technology Appraisal Guidance has been issued unless an extension has been authorised by the Secretary of State. In England, commissioning for orphan medicines falls within the remit of Specialist Commissioning.^1^ There are instances where NHS England has identified those services with small number of patients and high costs, such as provision of orphan medicines. These services would not be supplied at local level and instead patients are referred to regional or national treatment centres which specific funding arrangements. For the period under examinations, to June 2016, a number or OMPs for cancer indications were included within the Cancer Drugs Fund (CDF). This was a mechanism initially separated but subsequently included within the remit of Specialist Commissioning, allowing funding for cancer medicines either with a negative decision or not appraised by NICE.

HTA decisions by the SMC do not have mandatory funding but should be followed by NHS Boards and Area Drug and Therapeutic Committees (ADTCs) reimbursement decisions. Welsh Health boards have a legal requirement to implement NICE guidance and statutory funding directives apply with regards to NICE and AWMSG technology appraisals [17]. However, for the sake of clarity in the paper, we refer to all of these statutory funding decisions in the UK as OMPs being “reimbursed” while we exclude from reimbursement nominative prescriptions through Individual Funding Requests (IFR) in England and Wales and Individual Patient Treatment Requests (IPTR) in Scotland. We did not consider the OMPs with a negative recommendation by the SMC which could be potentially funded through the £40 m New Medicines Fund created in 2014 in Scotland because a detailed list was not available in the public domain.

In Spain and Italy, there are no specific departures from the standard pricing and reimbursement process for OMPs coverage decisions, although there are ear-marked research funds to cover OMPs in both countries (e.g. €17 m in 2013 in Italy, and €16 m in 2014 in Spain). Nonetheless, several Italian regulations and agreements have favoured P&R arrangements for OMPs while we are not aware of this case in Spain. In Spain, a national transposition of the EC authorisation takes place soon after the granting of the authorisation by the EC and always before the reimbursement. Most of the new innovative medicines authorised by the Spanish Medicines Agency (AEMPS) are distributed directly to hospitals and dispensed by the hospital pharmacy. The distribution to hospital pharmacies follows direct purchases by hospitals, frequently centralised at regional level, and including rebates [15], and little is known about prices or whether different regions and hospitals offer different OMPs.

In Italy, either the “local health authority” or the “region” can decide on reimbursement, except in the case of pre-authorisation use which is fully reimbursed under the national law 648 of 1996. This law allows reimbursement of pharmaceuticals for which no therapeutic alternative exists and some off-label use. Some OMPs are available before the price is negotiated between the producer and AIFA via the Cnn class. Hospital drugs are fully reimbursed in Italy.

## Methods

A good indicator of access to a medicinal product is the percentage of patients who are effectively treated within the public health service. We construct indicators to approximate this concept considering similar approaches in the literature ([5]; Le [4]).

We compare access to OMPs in the five major European countries (by pharmaceutical value): France, Germany, Italy, Spain, and the UK. For the UK, we compare access in England, Scotland, and Wales using information from the three HTA agencies in these nations. No information is included for Northern Ireland, as it does not have its own HTA agency.

### OMPs included in the analysis

We have included all EU centrally authorised OMPs since the inception of the orphan regulation in 2000 to end of May 2016. We obtained the list of OMPs authorised via the centralised procedure since 2001 from the European Medicines Agency’s (EMA) and DG Health and Food Safety’s websites. Given the large time span considered, for some of the OMPs included in the study the market exclusivity or the patent has expired (e.g. imatinib). This does not affect our analysis as we considered the reimbursement status near launch.

We have used the current community register of medicinal products authorised via the centralised procedure published by the EC, all the annexes of the Annual Reports published by the EMA since 2001 which contain a listing of the Committee for Medicinal Products for Human Use (CHMP) opinions on OMPs and their authorisations and the report “Inventory of Union and Member State incentives to support research into, and the development and availability of, orphan medicinal products. State of Play 2015” [7]. The time period covered since 2000, i.e. date of implementation of Regulation (EC) No 141/2000 on OMPs, to the 31st of May 2016, and the information extracted included the invented name, international non-proprietary name, orphan indication(s), date of EC decision (date of the marketing authorisation).

During this period the CHMP issued positive opinion to grant authorisation for 128 new substances, with 15 of them having also a successive extension/s of indication. Although most studies consider the number of OMPs at the substance level (e.g [2] report 129 centrally authorised OMPs), we identify each authorised OMPs by its central marketing authorisation wave. This definition of OMPs which splits substances for each new authorisation wave (extension of indication) is used in most HTA evaluations at country level and considers the dossier submitted by the manufacturer to EMA i.e. they are treated as de facto different products. Therefore, sorafenib corresponds to two OMPs (initial authorisation for hepatocellular carcinoma and extension of indication for thyroid carcinoma) and we consider 143 OMPs in total as potentially available to define our benchmark for comparison across the selected countries.

Data on orphan designations granted by EC between 2001 and the 31st of May 2016 were also collected and compared with the number of OMPs which achieve EC marketing authorisation.

### Definitions and indicators of availability and access

Availability was defined as the possibility that an OMP can be prescribed within the national health system and dispensed in pharmacies or hospitals (even if not reimbursed by the national health system) or is being used following clinical development, for example in early access schemes. The extraction of data on availability considered any decision issued by a national regulatory authority allowing the use of these OMPs in their territory. Some of the previous studies have identified this type of availability by reporting the number of OMPs marketed in the country [5, 6, 9, 11, 12, 16], even though a non-commercialised OMP can be made available to patients before central authorisation through early access schemes and compassionate use. Since we only have information on market launch of the OMPs for France, we consider the three following indicators to proxy availability understood as the number of OMPs marketed:

1. Whether the OMP has been registered in a national register which is a transposition of the central marketing authorisation. In Germany, and Spain, the available OMPs are those included in national registers, and this information corresponded in Italy to the date at which the first decision was published by the Italian Medicines Agency (AIFA).
2. Whether the OMP has pre-authorisation use. Data on pre-license use is only available for France and Italy, although the UK and Spain also have early access schemes and compassionate use programmes. In Italy, OMPs with pre-authorisation use are included in the AIFA register.
3. Whether there is a published HTA assessment for the OMP. Data on HTA decisions is publicly available for the HTA agencies in France, Germany, and the three UK nations. The information on HTA evaluations was used to define whether an OMP is available in the UK. In England, published HTA data was obtained from the National Institute for Health and Care Excellence (NICE) evaluation programmes (Technology Appraisal (TA) and Highly Specialised Technologies (HST)), and complemented with NHS England commissioning policies and inclusion in the Cancer Drugs Fund (CDF). For Scotland, the available OMPs are assumed to be those appraised by the Scottish Medicines Consortium (SMC), since the consortium has a mandate to evaluate all authorised medicines. The Welsh agency, All Wales Medicines Strategy Group (AWMSG) adopts NICE guidance where available and appraises the remaining new medicines not on the NICE work programme. Therefore, availability in Wales is restricted to these medicines with an evaluation published by AWMSG or NICE.

The second measure, “access”, was defined as a full or partial reimbursement of an OMP by the public healthcare system. Importantly, we consider access for all patients specified in the product marketing authorisation and interpret reimbursement through positive lists or routine funding and not as a case-by-case. Therefore, the information on reimbursement of OMPs was collected taking into account the different national reimbursement policies. For example, NICE makes recommendations concerning the funding (or not) of these medicines in the NHS which must be implemented by local commissioners. For the sake of clarity, we refer to all of these funding decisions as OMPs being “reimbursed”.

Details of P&R for each country have been summarised in section 2.

### Data sources and extraction

External experts were commissioned to extract data from Italy, Spain and to validate the data from Germany. The database was constructed from public information for each country. A complete list of data sources is included at the end in the reference section. We developed a protocol to ensure a systematic and consistent approach to data extraction for the UK HTA systems and the other four EU countries.

For some OMPs there were multiple HTA decisions in Scotland and England. In case of repeated submissions to the SMC, we considered the publication of a positive HTA decision with its publication dates, to define the time when reimbursement was possible in Scotland. NICE has also updated TA Guidance for the same OMP indication in three cases: ruxolitinib for primary myelofibrosis, bosutinib for chronic myeloid leukaemia, and trabectedin for recurrent ovarian cancer. For these OMPs we considered the last NICE decision and its publication date. Also, NICE published two different TAs, for first- and second-line, for the same therapeutic indication for two OMPs: sunitinib for metastatic renal-cell carcinoma and nilotinib for chronic myeloid leukaemia. Both are considered in our analysis.

Time to access was defined as the time elapsed between EC authorisation and date of inclusion in the relevant positive list; the date of the publication of an HTA recommendation, of a P&R or commissioning decision, depending on the country.

Table 1 presents the definition of the indicator of availability and access for each country. The consideration of available OMPs through HTA decisions as in the three UK devolved nations needs a further clarification. For example, available OMPs in Scotland are all those which have a register to be evaluated by SMC, including instances where the manufacturer does not submit a dossier where SMC issues a “not recommended” decision. Non-submissions are also included as HTA evaluations for NICE and AWMSG with a decision of “unable to recommend”. For other HTA evaluations, the respective agency can issue a positive decision: either “recommended” within the marketing authorisation, or “optimised” which restricts the group of patients specified by the marketing authorisations. A negative decision is “not recommended”. The HTA positive decisions are considered to conduce to access to OMPs in the UK devolved nations.

**Table 1:** List of all extracted information from EMA and each country matched with the definition of indicators for availability and access

Country	Indicators	Data sources (listed in Appendix)	Indicator definition
EC	Central marketing authorisation	European Medicines Agency	• Benchmark of potentially available OMPs. • Central marketing authorisation date used to define time to access indicator in each country.
France	Early access schemes	ANSM – Autorisations temporaires d’utilisation	Reported number of OMPs to complement indicator of availability.
	Launch	Base de données publique des medicaments	Availability = Number of OMPs.
	HTA	Base de données publique des medicaments HAS	Reported number of OMPs with HTA evaluation and decision to complement indicator of access.
	Reimbursement	Base de données publique des medicaments Base Vidal HAS	• Access = Number of OMPs reimbursed. • Time to access = reimbursement date (normally after HTA publication date, except for some pre-license OMPs) - central authorisation date.
Germany	National registry/authorisation	Lauer-Taxe database	• Availability = Number of OMPs. • Access = Number of OMPs. • Time to access = 0
	HTA	IQWiG G-BA	Reported to complement and interpret P&R policies.
Italy	Early access schemes	AIFA list of drugs	Reported and included in availability indicator.
	National registry/authorisation	AIFA list of drugs (includes pre-licensed OMPs)	Availability = Number of OMPs.
	Reimbursement	Manual consultación of the “Gazzetta Ufficiale” and other relevant laws such as Law 648/1996	• Access = Number of OMPs reimbursed. • Time to access = reimbursement date (publication Gazetta Ufficiale) – central authorisation date.
Spain	National registry/authorisation	Consejo General de Colegios Oficiales de Farmacéuticos	Availability = Number of OMPs.
	Reimbursement	Consejo General de Colegios Oficiales de Farmacéuticos	• Access = Number of OMPs. • Time to access = reimbursement date (date enter in reimbursement registry) – central authorisation date.
England	HTA Other routine funding	NICE (Technology Appraisals and Highly Specialised Technologies) NHS England (reimbursement date not available) CDF (reimbursement date not available)	• Availability = Number of OMPs (publication of NICE guidance or other routine funding list). • Access = Number of OMPs (positive HTA decision (including treatment recommended for use, optimised), or commissioning policy in other routine funding list). • Time to access = HTA publication date – central authorisation date.
Scotland	HTA	SMC	• Availability = Number of OMPs with HTA evaluation. • Access = Number of OMPs with positive HTA decision (including treatment recommended for use, optimised). • Time to access = HTA publication date – central authorisation date.
Wales	HTA	NICE AWMSG	• Availability = Number of OMPs with HTA evaluation. • Access = Number of OMPs with positive HTA decision. • Time to access = HTA publication date – central authorisation date.

### Analysis

We present descriptive analysis statistics to compare availability and access to OMPs. For the indicator of time elapsed between EC authorisation and reimbursement, we assess between-country differences by presenting the time-to-access curves (Kaplan-Meier curves) which describe the complement of the cumulative frequency of reimbursed OMPs in each country, except Germany, from time zero of immediate reimbursement (100% of OMPs not reimbursed) to maximum delayed time of reimbursement (all OMPs reimbursed or 0% remain to be reimbursed). Therefore, the Kaplan-Meier curve informs the speed of reimbursement within a country (e.g. whether the majority of OMPs are reimbursed in a short or long period, corresponding to the median (50%), or about the percentage of OMPs reimbursed with a very long delay) and this can be compared across countries.

## Results

### OMPs with an EC marketing authorisation

Since 2000, when the Orphan Regulation came into force, up to end May 2016, the EC granted marketing authorisation to 143 OMPs. This represents an approval rate of 10.5% over the 1360 orphan designations. Of the total number of OMPs with marketing authorisation, nearly 40% (56) were indicated for oncology. Table 2 shows how the approval rate varies slightly over time although both the number of designations and authorisations increased. The time interval between orphan designation and marketing authorisation averaged 4 years and 7 months for the whole period. This interval has decreased over time and is just over 15 months for the OMPs which were authorised in 2014.

**Table 2:** Orphan Designations and Marketing Authorisations

	2001–2005	2006–2010	2011	2012	2013	2014	2015	2016 (Jan-May)	Total
Number of OMPs designations	173	355	86	118	124	182	185	137	1360
Number of OMPs authorised	22	45	7	12	10	17	20	10	143
% OMPs Designations ^a^	12.7%	12.7%	8.1%	10.2%	8.1%	9.3%	10.8%	7.3%	10.5%
Average months elapsed from designation to authorisation ^b^	72.7	54.0	47.1	29.4	19.9	15.6	n/a	n/a	54.7

Source: Authors’ elaboration from data available at EMA website

^a^This percentage denotes the proportion of OMPs authorised compared to the number of designations granted by the EC during the same period of time.

^b^The computation of months from designation to authorisation is specific to the product, the last orphan designation which was authorised was designated in 2014 (but authorised in later years)

**Fig. 1:**
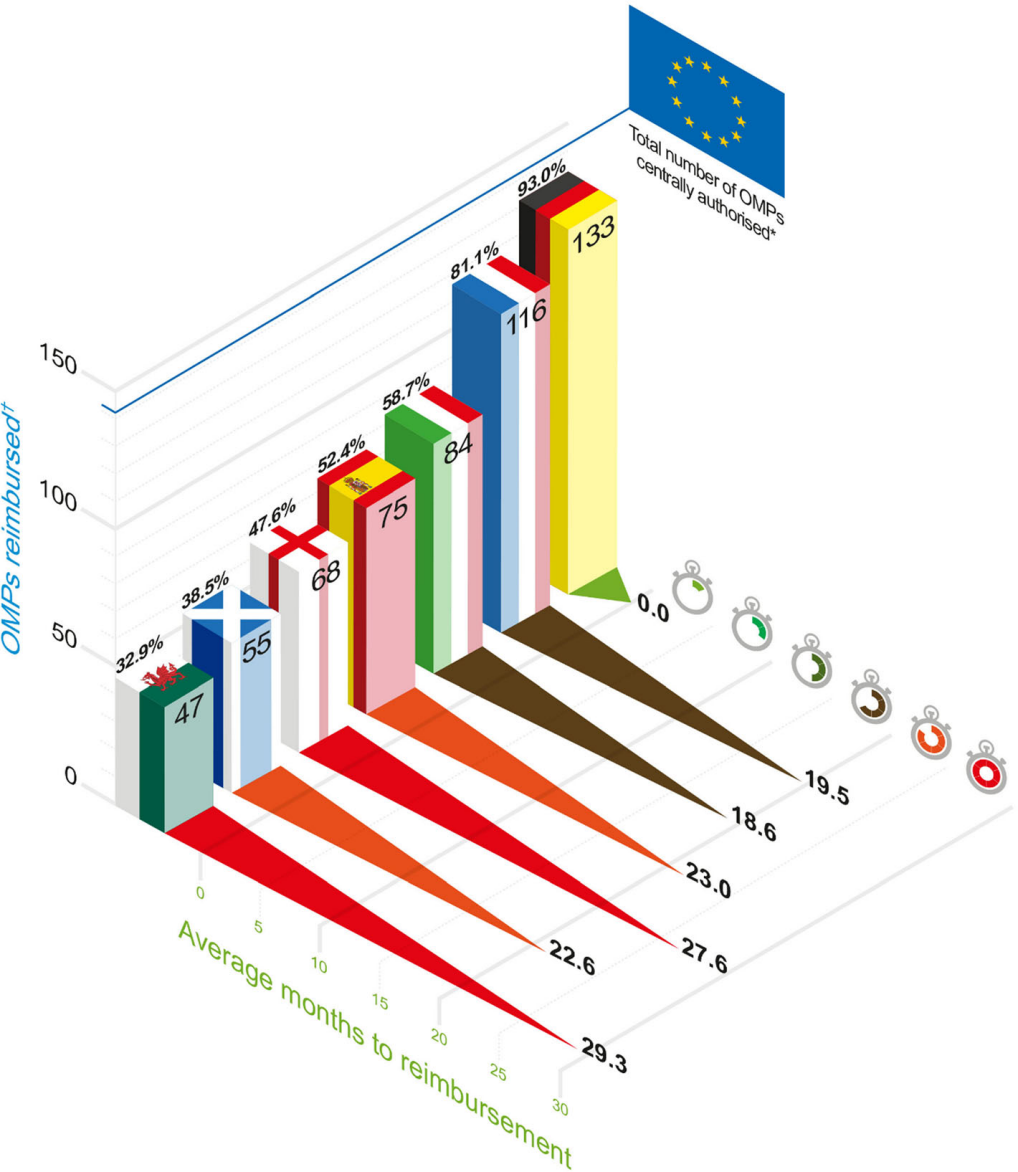
Comparison of access to OMPs. Notes: * 143 OMPs obtained a marketing authorisation since the implementation of the EU Regulation on Orphan. Medicines (Regulation (EC) No 141/2000). † OMPs reimbursed refers to Health Technology Assessment (HTA) recommendations to use or inclusion in reimbursement lists in respective national health systems

Figure 1 shows that the authorised 143 OMPs are most widely accessible in Germany (93%) and France (81%).

In the German system, 134 commercialised OMPs are included in the Lauer-Taxe database; all of them but one^2^ are fully reimbursed since their entry in the German market. Since AMNOG introduction (2011), there have been 45 assessments of OMPs published, with 37 of them completed and ranking therapeutic value according to “additional benefit” which affected price negotiation and rebates.

### Country by country analysis on availability and access to OMPs

In France, pre-authorisation through Temporary Authorisation for Use (ATUs) explains the larger number of OMPs reimbursed (116) than those marketed (108). A total 119 HTA evaluations have been issued for OMPs. In particular, 15 OMPs with a HTA evaluation had not been launched at the time of the analysis. Conversely, 4 OMPs have been marketed but do not have any evaluation by the HAS. This includes daratumumab (Darzalex), which was only recently authorised (May 2016) and it is reimbursed in absence of HTA evaluation.

In Italy, 125 OMPs are included in the registry by the AIFA which follows the EC marketing authorisation in most cases, but there are instances where the AIFA published a decision before the granting of the marketing authorisation. Additionally, there is a pre-authorisation programme and all OMPs used in the early access scheme have systematically been registered by AIFA.

According to the information on drug reimbursement decisions published in the “Gazzetta Ufficiale” (Italian Official Journal), 84 OMPs (58.7% of centrally authorised OMPs) are reimbursed by the national health system. There is availability of 41 OMPs authorisation via an early access scheme, and all of these are included in the AIFA register and are reimbursed.

In Spain, only 79 OMPs have been included in the AEMPS (Spanish Agency of Medicinal Products and Medical Devices) register. There is no information on availability through pre-license programmes although medicines under clinical development can be used via compassionate use programmes since 2009.

All but 4 registered OMPs (75 out of 79) are reimbursed, which represents a 52.4% of centrally authorised OMPs. Most OMPs are hospital prescribed and fully reimbursed.

UK presents a percentage of OMPs reimbursed between 32.9 and 46.9%. Comparing the three UK nations, Table 3 shows that there is greater availability and accessibility of orphan medicines in England where most of the 68 OMPs were reimbursed because they were included in the NHS England specialised commissioning list (32) or the Cancer Drugs Fund (13). NICE positive decisions (including recommended for use within the marketing authorisation, and “optimised” which applies some restrictions to the marketing authorisation) were observed for 23 OMPs (43.4%). In contrast, in Scotland and Wales we found higher proportion of positive HTA decisions (57.3% in Scotland, and 55.9% in Wales, which includes adoption of NICE decisions by AWMSG). An analysis of recommendations given by AWMSG between 2002 and 2014 reported a similar percentage of positive recommendations (59% for orphan medicines and 73% of ultra-orphan, as cited in [17]).

**Table 3:** Numbers and percentages of Available and Accessible OMPs

	Available OMPs				Reimbursed OMPs	
	Pre-authorisation		Decision on use^a^			
	Total number	% out of 143	Total number	% out of 143	Total number	% out of 143
France	83	58.0%	108	75.5%	116	81.1%
Germany	–	–	134	93.7%	133	93%
Italy	41	28.7%	125	87.4%	84	58.7%
Spain	–	–	79	54.2%	75	52.4%
England	–	–	120	82.8%^b^	68	46.9%^b^
Scotland	–	–	96	67.1%	55	38.5%
Wales	–	–	84	58.7%	47	32.9%

Source: Authors’ elaboration from data listed in references and indicators defined in Table 1.

^a^Number of available OMPs correspond to those with information of a “decision on use” which is defined in Table 1: OMPs marketed in France; OMPs included in national transpositions of EC authorisation registries for Germany, Italy, and Spain; OMPs referred to HTA evaluation in Scotland and Wales; OMPs listed in the NHS England drugs list.

^b^For these 143 OMPs, 145 different indications were appraised by NICE in England (split indications for sunitinib and nilotinib). Reported percentages for England were calculated over 145 indications.

### Country by country analysis on time to access of OMPs

**Fig. 2:**
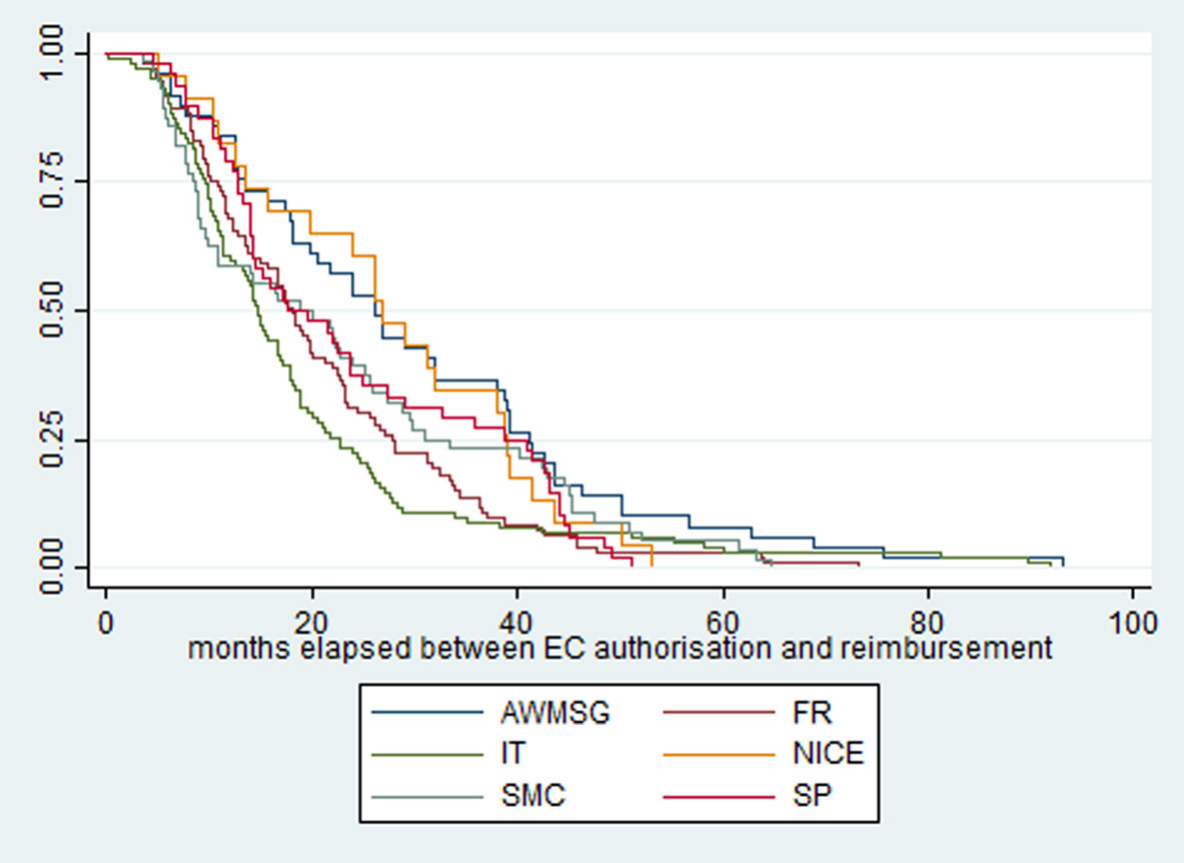
Kaplan-Meier curves of time-to-access

Table 4 presents descriptive statistics of the time elapse between EC marketing authorisation an reimbursement in each selected country. These statistics are complemented with a graphical description of time-to-access curve in Fig. 2. When analysing the time to access presented in Fig. 2 and Table 4, we observe that in England the maximum time from authorisation to publication of positive HTA recommendations was less than 53 months while Wales and Italy had longer delays for around 10% of OMPs. In Scotland we observed the quickest time to access 75% of OMPs are provided quicker than in England.

**Table 4:** Comparison of months elapsed between EC authorisation and reimbursement/publication of HTA recommendation

	Minimum	Maximum	Median	Mean
France	−73.6^a^	76.2	17.5	19.5
Italy	−3.0^a^	92.0	14.5	18.6
Spain	4.5	51.1	17.4	23.0
England	5.1	53.2	26.8	27.6
Scotland	3.6	64.6	18.8	22.6
Wales	3.6	93.1	26.2	29.3

Source: Authors’ elaboration from data listed in references and time-to access indicator defined in Table 1.

^a^Three OMPs were reimbursed in France before EC authorisation: carglumic acid (73.6 months), ataluren (1.3 month), temsirolimus (18 months). One OMP was reimbursed in Italy before EC authorisation: susoctocog alfa (3 months).

Excluding Germany, Italy is the country with the most rapid reimbursement decisions. Only two decisions experienced a long delay of about 90 months (nitisinone and eculizumab). In terms of average delay, France and Italy present shorter delays which are affected by reimbursement through pre-authorisation schemes. For example, 3 pre-authorised OMPs were reimbursed in France prior to obtaining EC authorisation.

## Discussion

The indicators we adopted to measure availability and access to OMPs have a series of limitations. The publication of HTA reimbursement decisions does not guarantee the actual availability of medicines to patients. This is for two reasons. Firstly, new medicines are not necessarily subject to an HTA process, therefore our measure of availability might underestimate the number of OMPs launched and potentially available to patients. Secondly, some HTA agencies conduct evaluations involving OMPs that the marketing authorisation holder will subsequently decide not to market in this country, although this was only observed in France. This might lead to an overestimation of OMPs available.

There are a number of mechanisms other than HTA approvals and P&R decisions by which patients can access OMPs. These include open extensions of pivotal clinical trials or compassionate programmes put in place and funded by marketing authorisation holders, early access schemes put in place by national health authorities (e.g. France, UK) and individual patient funding requests (e.g. in the UK). Apart from data on ATU in France and early access schemes in Italy, our data do not capture those alternative routes of funding in the national health systems and so might underestimate the actual number of OMPs that patients received.

One limitation of using HTA decisions as a proxy of access is that HTA positive recommendations do not necessarily lead to faster access to new medicines in practice since the recommendation needs to be implemented by local commissioning bodies (e.g. in the UK). For example, there is a 3-month time-period in the provision of statutory funding following a NICE recommendation. Since AWMSG adopts NICE decisions, a longer delay is observed in AWMSG than NICE decisions. Varnava et al. [17] argue that the time-lag due to the manufacturer submitting first to NICE makes the AWMSG process appear longer when measured from marketing authorisation to recommendation. Germany has the shortest delay from the authorisation to the availability and access, taking into account that reimbursement decisions are automatic, with no delay from marketing authorisation. Yet Detiček et al. [6] report a median delay of three months between marketing authorisation and first continuous use from observed sales.

As we could expect, time to access to OMPs in clinical practice is shorter in the countries which have implemented early access schemes. This time is longer in countries where the implementation of HTA decisions is delayed (e.g. because of the budget impact of the medicine or price negotiations). Since the time to access new OMPs is affected by numerous factors (including companies ultimately deciding to delay the launch of a medicine in any particular country because of the use of international reference prices, where launch prices across countries are interlinked), more research is required to collect reliable data on uptake OMPs in clinical practice and assess the impact of factors driving it.

Another limitation is that the time elapsed between EU marketing authorisations included in our analysis and the dates of marketing in individual countries were not captured since our data sources do not include company statements on the dates of marketing of these products.

Our analysis was based on publicly available information. In some instances (in Spain), managed entry agreements are negotiated at regional level. Very little public information is available on these agreements or on centralised hospital purchases. Equally, information on reimbursement is not published at regional level in Italy. Only national decisions by the AIFA are published in the official gazette joint with the publication of all laws. For Spain and Italy we only considered data produced at the national level since regional HTA do not provide data systematically. For example, only 7 out of 17 Spanish regions have an HTA agency, and web-available reports are only available in 4 Italian HTA agencies, although are in principle devolved to the 20 Italian regions [8].

Compared to the published literature in the topic, there are some convergence in the results but also some discrepancies.

Compared to the most recently published analysis, Detiček et al. [6], there is an alignment on the results on Germany as the country with widest access to OMPs. However, Detiček et al. present UK as a country with wider access than Italy, France, and Spain. We justify this difference with the data used to measure access, which is the observation of positive sales in the case of Detiček et al.’s. Our choice was driven by availability of data in the public domain (as opposed to sales data) in all selected countries and also, conceptually, by the role of assessment (including HTA) and P&R processes to signal the ability and willingness to pay for individual medicines by national health systems to all patients as specified in the marketing authorisation.

Our study is aligned with the main results of other previous studies, with France and/or Germany at the top of the accessibility ranking based on different indicators: reimbursement ([3, 5, 9]; Le [4]), and market sales [12].

Considering the ranking or access to OMPs among the five selected countries some contradictions also exist in the literature. We have already argued that one key factor to explain these contradictions is the definition of the access indicators, either via P&R decisions or sales data. Other factor is the benchmark considered to define the set of potentially available OMPs. Several studies present differences with the ranking of access we present. For example, Orofino et al. [11] and Detiček et al. [6] coincide in placing Germany followed by the UK according to a definition of access based on sales data, contradicting then our finding of lower access for the UK devolved nations. The position of France is also sensitive to the different definition of access indicator, arguably due to the different consideration of early access schemes in France, with no sales data.

The literature also presents some different interpretations of the role of HTA on P&R decisions, in particular for France and Germany. A recent study [10] found France providing reimbursement to 20% of OMPs, which is lower than the percentage of positive HTA decisions in France reported by the same authors. For Germany, Kawalec et al. [10] report a low rate of HTA assessments and reimbursement which does account neither for the late introduction of HTA in 2011 nor for the fact that HTA is not a pre-requisite for reimbursement in Germany [1] in the sense we have explained in section 2. Overall, our study presents a comprehensive selection of OMPs covering over 15 years access to OMPS since the inception of the EU orphan drug regulation in 2000.

## Conclusions

Our study suggests that one of the intended effects of this regulation, to grant equal availability to OMPs to patients in the EU via the implementation of the OMPs Regulation, was partially achieved with important variations in availability and access across the selected countries. We found that more than a half of centrally authorised OMPs were available in the five selected countries, but that access to patients was further restricted by different national reimbursement policies, especially in the UK, Italy and Spain. Our study focused on the five largest economies of the 28 EU member states. It highlighted the potential role of a number of national policies, such as the extended remit to HTA in France facilitating pre-authorisation access, and a two-stage administered system in Germany which prioritises reimbursement over price negotiations. More research is recommended to collect reliable data on use of OMPs in clinical practice and assess the impact of individual policies (orphan and non-orphan specific) on access.

### Additional file

Additional file 1: Presents a table with the list of 143 OMPs with relevant EC authorisation data matched with a reimbursement binary indicator for each country. (XLSX 28 kb)

## Appendix

### European Medicines Agency and European Commission

European Medicines Agency, European Public Assessment Reports of Centrally authorised medicinal products, orphan designations recommendations and annual reports.

DG Health and Food Safety (community registers of orphan medicinal products for human use and medicinal products for human use) [Accessed September 2016].

Regulation (EC) No 141/2000 of the European Parliament and of the Council of 16 December 1999 on orphan medicinal products, O.J. L 18, 22.1.2000.

Regulation (EC) No 726/2004 of the European Parliament and of the Council laying down Community procedures for the authorisation and supervision of medicinal products for human and veterinary use and establishing a European Medicines Agency, OJ L 136, 30.4.2004.

### France

Base de données Publique des Médicaments:

http://base-donnees-publique.medicaments.gouv.fr/ [Accessed September 2016].

Base de données Vidal des medicaments.

https://www.vidal.fr/Sommaires/Medicaments-A.htm [Accessed September 2016].

Haute Autorité de la Santé (HAS).

http://www.has-sante.fr/portail/ [Accessed September 2016].

Official Journal.

https://www.legifrance.gouv.fr/initRechTexte.do;jsessionid=98B5C38BE58B2BE9A49767469A3FC173.tpdila15v_1 [Accessed September 2016].

Agence Nationale de Securité des Médicaments (ANSM) – Autorisations temporaires d’utilisation.

http://ansm.sante.fr/Activites/Recommandations-Temporaires-d-Utilisation-RTU/Les-Recommandations-Temporaires-d-Utilisation-Principes-generaux/(offset)/0 [Accessed September 2016].

Ministere des affaires sociales et de la sante.

http://social-sante.gouv.fr/soins-et-maladies/medicaments/professionnels-de-sante/prescription-et-dispensation/article/medicaments-retrocedes-retrocession [Accessed September 2016]

### Germany

Institute for Quality and Efficiency in Health care (IQWiG).

https://www.iqwig.de/en/home.2724.html [Accessed September 2016].

Federal Joint Committee (G-BA).

http://www.english.g-ba.de/benefitassessment/information/ [Accessed September 2016].

Lauer-Taxe database: https://www.lauerfischer.de/LF/Seiten/Verwaltung/Kundencenter/1.aspx [Accessed September 2016]

### Italy

Agenzia Italiana del Farmaco (AIFA).

http://www.aifa.gov.it/en [Accessed September 2016].

AIFA list of drugs: http://www.agenziafarmaco.gov.it/it/content/lista-aggiornata-dei-registri-e-dei-piani-terapeutici-web-based [Accessed September 2016].

Manual consultación of the “Gazzetta Ufficiale” and other relevant laws such as Law 648/1996

### Spain

Spanish Agency of Medicinal Products and Medical Devices.

https://www.aemps.gob.es/en/home.htm [Accessed September 2016].

Consejo General de Colegios Oficiales de Farmacéuticos https://botplusweb.portalfarma.com/ [Accessed September 2016]

### United Kingdom

NICE (National Institute for Health and Care Excellence).

https://www.nice.org.uk/ [Accessed September 2016].

NHS England (Indications for NHS England drugs list).

https://www.england.nhs.uk/commissioning/wp-content/uploads/sites/12/2016/12/nhs-england-drugs-list-v11.pdf [Accessed in September 2016 nhs-england-drugs-list-v10.pdf].

SMC (Scottish Medicines Consortium).

https://www.scottishmedicines.org.uk/ [Accessed September 2016].

AWMSG (All Wales Medicines Strategy Group).

http://www.awmsg.org/ [Accessed September 2016]

## Abbreviations

AEMPS: Spanish Agency of Medicinal Products and Medical Devices
AIFA: Italian Medicines Agency
ALD: Affections de Longue Durée
AMNOG: Act on the Reform of the Market for Medical Products
ASMR: Amélioration du Service Médical Rendu
ATU: Temporary Authorisation of Use
AWMSG: All Wales Medicines Strategy Group
CHMP: Committee for Medicinal Products for Human Use
EC: European Commission
EMA: European Medicines Agency
EU: European Union
EURORDIS: European Organisation for Rare Diseases
G-BA: Federal Joint Committee
HAS: Haute Autorité de la Santé
HST: Highly Specialised Technologies
HTA: Health Technology Assessment
IQWiG: Institute for Quality and Efficiency in Health Care
NHS: National Health Service
NICE: National Institute for Health and Care Excellence.
OMP: Orphan Medicinal Product
P&R: Pricing and Reimbursement
SMC: Scottish Medicines Consortium
SMR: Service Médical Rendu
TA: Technology Appraisal

## Footnotes

1
https://www.england.nhs.uk/commissioning/spec-services/

2 Note bedaquiline which is indicated for pulmonary multidrug resistant tuberculosis (MDR TB), has been excluded from the reimbursement list due to not meeting package size regulation.

## References

[1] Akehurst RL, Abadie E, Renaudin N, Sarkozy F (2017). Variation in health technology assessment and reimbursement processes in Europe. Value Health.

[2] Annemans L, Aymé S, Le Cam Y, Facey K, Gunther P, Nicod E, Reni M, Roux J-L, Schlander M, Taylor D (2017). Recommendations from the European working Group for Value Assessment and Funding Processes in rare diseases (ORPH-VAL). Orphanet J Rare Dis.

[3] Bignami F (2007). Eurordis survey on orphan drugs availability in Europe.

[4] Le Cam, Y., 2011. Inventory of access and prices of orphan drugs across Europe. https://img2.eurordis.org/newsletter/pdf/mar-2011/ERTC_13122010_YLeCam_Final.pdf. Last accessed 17 Sep 2011.

[5] de Varax A, Letellier M, Börtlein G (2015). Study on orphan drugs: phase I: overview of the conditions for marketing orphan drugs in Europe.

[6] Detiček A, Locatelli I, Kos M (2018). Patient access to medicines for rare diseases in European countries. Value Health..

[7] European Commission, 2016. Inventory of Union and Member State incentives to support research into, and the development and availability of, orphan medicinal products. State of play 2015.

[8] Garattini L, van de Vooren K, Curto A (2012). Regional HTA in Italy: promising or confusing?. Health Policy.

[9] Garau, M. and J. Mestre-Ferrandiz, 2009. Access mechanisms for orphan drugs: a comparative study of selected European countries.

[10] Kawalec P, Sagan A, Pilc A (2016). The correlation between HTA recommendations and reimbursement status of orphan drugs in Europe. Orphanet J Rare Dis.

[11] Orofino J, Soto J, Casado MA, Oyagüez I (2010). Global spending on orphan drugs in France, Germany, the UK, Italy and Spain during 2007. Appl Health Econ Health Policy.

[12] Picavet E, Annemans L, Cleemput I, Cassiman D, Simoens S (2012). Market uptake of orphan drugs–a European analysis. J Clin Pharm Ther.

[13] Stolk P, Heemstra HE, Leufkens HG, Bloechl-Daum B, Heerdink ER (2009). No difference in between-country variability in use of newly approved orphan and non-orphan medicinal products-a pilot study. Orphanet J Rare Dis.

[14] Tordrup D, Tzouma V, Kanavos P (2014). Orphan drug considerations in health technology assessment in eight European countries. Rare Dis Orphan Drugs.

[15] Towse A, Cole A, Zamora B. The debate on indication-based pricing in the U.S. and five major European countries. OHE Consulting Report, London: Office of Health Economics; 2018. Available at: https://www.ohe.org/publications/debate-indicationbased-pricing-us-and-five-major-european-countries.

[16] Trama A, Pierannunzio D, Loizzo A, Taruscio D, Ceci A (2009). Availability of medicines for rare diseases in EU countries. Pharmaceuticals Policy Law.

[17] Varnava A, Bracchi R, Samuels K, Hughes DA, Routledge PA (2018). New medicines in Wales: the all Wales medicines strategy group (AWMSG) appraisal process and outcomes. PharmacoEconomics.

